# Non-ictal, interictal and ictal déjà vu: a systematic review and meta-analysis

**DOI:** 10.3389/fneur.2024.1406889

**Published:** 2024-06-20

**Authors:** Alena Hadzic, Stein Andersson

**Affiliations:** ^1^Section for Clinical and Cognitive Neuroscience, Department of Psychology, University of Oslo, Oslo, Norway; ^2^Section for Pharmacology and Pharmaceutical Biosciences, Department of Pharmacy, University of Oslo, Oslo, Norway; ^3^Psychosomatic Medicine and CL Psychiatry, Division of Mental Health and Addiction, Oslo University Hospital, Oslo, Norway

**Keywords:** epilepsy, seizure, non-ictal, interictal, ictal, déjà vu, systematic review, meta-analysis

## Abstract

**Background:**

Déjà vu, French for “already seen,” is a phenomenon most people will experience at least once in their lifetime. Emerging evidence suggests that déjà vu occurs in healthy individuals (as “non-ictal déjà vu”) and in epilepsy patients during seizures (as “ictal déjà vu”) and between seizures (as “interictal déjà vu”). Although the ILAE has recognized déjà vu as a feature of epileptic seizures, it is notably absent from the ICD-11. A lack of evidence-based research may account for this omission. To our knowledge, this study represents the first systematic review and meta-analysis on déjà vu experiences. Through detailed examinations of non-ictal, interictal and ictal déjà vu, we seek to highlight possible clinical implications. Rethinking the status quo of ictal déjà vu could potentially lead to earlier interventions and improve outcomes for epilepsy patients.

**Methods:**

This study was registered in PROSPERO (ID: CRD42023394239) on 5 February 2023. Systematic searches were conducted across four databases: EMBASE, MEDLINE, PsycINFO, and PubMed, from inception to 1 February 2023, limited to English language and human participants. Studies were included/excluded based on predefined criteria. Data was extracted according to the PICO framework and synthesized through a thematic approach. Meta-analyses were performed to estimate prevalence’s of the phenomena. Study quality, heterogeneity, and publication bias were assessed.

**Results:**

Database searching identified 1,677 records, of which 46 studies were included. Meta-analyses of prevalence showed that non-ictal déjà vu was experienced by 0.74 (95% CI [0.67, 0.79], *p* < 0.001) of healthy individuals, whereas interictal déjà vu was experienced by 0.62 (95% CI [0.48, 0.75], *p* = 0.099) and ictal déjà vu by 0.22 (95% CI [0.15, 0.32], *p* = 0.001) of epilepsy patients. Examinations of phenomenological (sex, age, frequency, duration, emotional valence, and dissociative symptoms) and neuroscientific (brain structures and functions) data revealed significant variations between non-ictal, interictal and ictal déjà vu on several domains.

**Conclusion:**

This systematic review and meta-analysis do not support the notion that non-ictal, interictal and ictal déjà vu are homogenous experiences. Instead, it provides insight into ictal déjà vu as a symptom of epilepsy that should be considered included in future revisions of the ICD-11.

**Systematic Review Registration:**

https://www.crd.york.ac.uk/prospero/display_record.php?RecordID=394239, CRD42023394239.

## 1 Introduction

Déjà vu, French for “already seen,” is a widely recognized and intriguing phenomenon most people will experience at least once in their lifetime (1). Despite initial documentation dating back to A.D. 400 (2), the formal term “déjà vu” (3) and its definition as “any subjectively inappropriate impression of familiarity of the present experience with an undefined past” (4) were articulated two millennia later. Historically viewed through a mystical lens (5, 6), scientific efforts have sought to demystify déjà vu and empirically study it using various methods, including surveys, interviews, neuroimaging techniques, and performance-based neurocognitive assessments. This body of research indicates that déjà vu occurs in both non-clinical (7, 8) and clinical populations (9–12), particularly among epilepsy patients (13, 14).

Epilepsy is a brain disease characterized by a tendency to generate epileptic seizures (15), which are transient episodes of signs and/or symptoms caused by abnormal neuronal activity in the brain (16). Depending on the affected brain areas, seizures can be categorized as focal, generalized or unknown onset, involving motor or non-motor features, with or without impaired awareness (17). Epilepsy patients who report déjà vu as part of their seizure semiology are more likely to have focal seizures than other types of seizures (14). Emerging evidence suggests that these patients may experience two distinct forms of déjà vu: one during seizures and another between seizures (18). Despite the International League Against Epilepsy (ILAE) recognizing déjà vu as a feature of epileptic seizues (17), its diagnostic relevance is not fully acknowledged, notably absent from the International Statistical Classification of Diseases and Related Health Problems, Eleventh Revision (ICD-11) (19). A lack of evidence-based research may account for this omission.

This systematic review and meta-analysis aims to summarize current knowledge on déjà vu by comparing its occurrence in healthy individuals with that in epilepsy patients. For clarity, déjà vu in healthy individuals will be referred to as “non-ictal déjà vu.” Conversely, déjà vu in epilepsy patients will be referred to as “ictal déjà vu” when occurring during seizures and “interictal déjà vu” when occurring between seizures. By examining the phenomenological and neuroscientific aspects of non-ictal, interictal and ictal déjà vu, we seek to broaden our understanding and highlight potential clinical implications. Rethinking the status quo of ictal déjà vu could potentially lead to earlier interventions and improve outcomes for those predisposed to or diagnosed with epilepsy.

## 2 Methods

This study was registered in the International Prospective Register of Systematic Reviews (PROSPERO; ID: CRD42023394239) on 5 February 2023, with a predefined protocol. However, the unexpected breadth and depth of the literature encountered necessitated amendments to the original protocol. These amendments, aimed at refining our approach to ensure a focused and thorough analysis, included narrowing the clinical population, expanding the synthesis to incorporate meta-analyses, and adopting a suitable tool for quality assessment. Our methodology adhered to the Preferred Reporting Items for Systematic Reviews and Meta-Analyses (PRISMA) guidelines (20) and the recommendations outlined in the Cochrane Handbook for Systematic Reviews of Interventions (21). The review process was conducted independently by the authors, with any discrepancies encountered resolved through a consensus-based approach, ensuring the integrity and rigor of our findings.

### 2.1 Study search

Systematic searches were conducted across four electronic databases: EMBASE, MEDLINE, PsycINFO (all via Ovid), and PubMed, covering the period from inception to 1 February 2023. Our search strategy utilized the term “déjà vu” with the following syntax: “déjà vu” ([MeSH Terms] OR (“déjà” [All Fields] AND “vu” [All Fields]) OR “déjà vu” [All Fields]). We limited our searches to studies published in English and involving human participants.

### 2.2 Study selection

The study selection process included several stages: initially, titles and abstracts were screened for relevance; subsequently, full texts were assessed for eligibility; and finally, studies were included or excluded based on predefined selection criteria. The inclusion criteria comprised peer-reviewed empirical studies of various designs (randomized controlled trial (RCT), cohort, cross-sectional, case–control, case series, and case report), involving non-clinical (healthy) individuals and clinical (epilepsy) patients, examining non-ictal, interictal and ictal déjà vu, without restrictions on interventions/investigations or outcomes. The exclusion criteria, however, specifically eliminated studies examining analog déjà vu experiences.

### 2.3 Data extraction

Data extraction was performed employing the population, intervention/investigation, control, and outcome (PICO) framework, designed to support evidence-based practice (22).

### 2.4 Data synthesis

Qualitative and quantitative outcomes were synthesized through a thematic approach, focusing on the phenomenological aspects of non-ictal, interictal and ictal déjà vu, such as demographics, prevalence, frequency, duration, affect, and dissociation (derealization and depersonalization), as well as the brain structures and functions underlying these experiences. Sufficient numerical data were subjected to statistical analysis.

### 2.5 Quality assessment

The quality assessment of included studies was conducted using the Mixed Methods Appraisal Tool (MMAT) version 18 (23, 24). This tool evaluates the risk of bias across various empirical study designs, including qualitative studies, quantitative RCTs, quantitative non-randomized studies (NRS), quantitative descriptive studies, and mixed methods studies. An algorithm determined the specific category of study design. Each study was evaluated on five methodological quality criteria tailored to the quantitative descriptive study design, including relevance of sampling strategy, representativeness of sample, appropriateness of measurements, risk of nonresponse bias, and appropriateness of statistical analysis. Responses to these criteria were scored as 1 (“yes”), 0 (“no”), or ? (“cannot tell”), with affirmative responses being aggregated to generate an overall quality score ranging from 1 (20%) to 5 (100%).

### 2.6 Statistical methods

Meta-analyses were conducted to estimate the prevalence of non-ictal, interictal and ictal déjà vu, defined as the proportion of cases in a population (25). A random-effects model, using the DerSimonian and Laird method for between-study variance (26), was applied due to expected variability in proportions (27). Heterogeneity was evaluated using *I^2^*-statistics, categorized as low (< 40%), moderate (30–60%), substantial (50–90%), or considerable (75–100%) (27), and Cochran’s *Q*-test (28). Publication bias was assessed using Egger’s test (29). All statistical analyses were conducted with the “metafor” package (30) in RStudio (31), considering *p*-values <0.05 as statistically significant.

## 3 Results

### 3.1 Study selection

**Figure 1:**
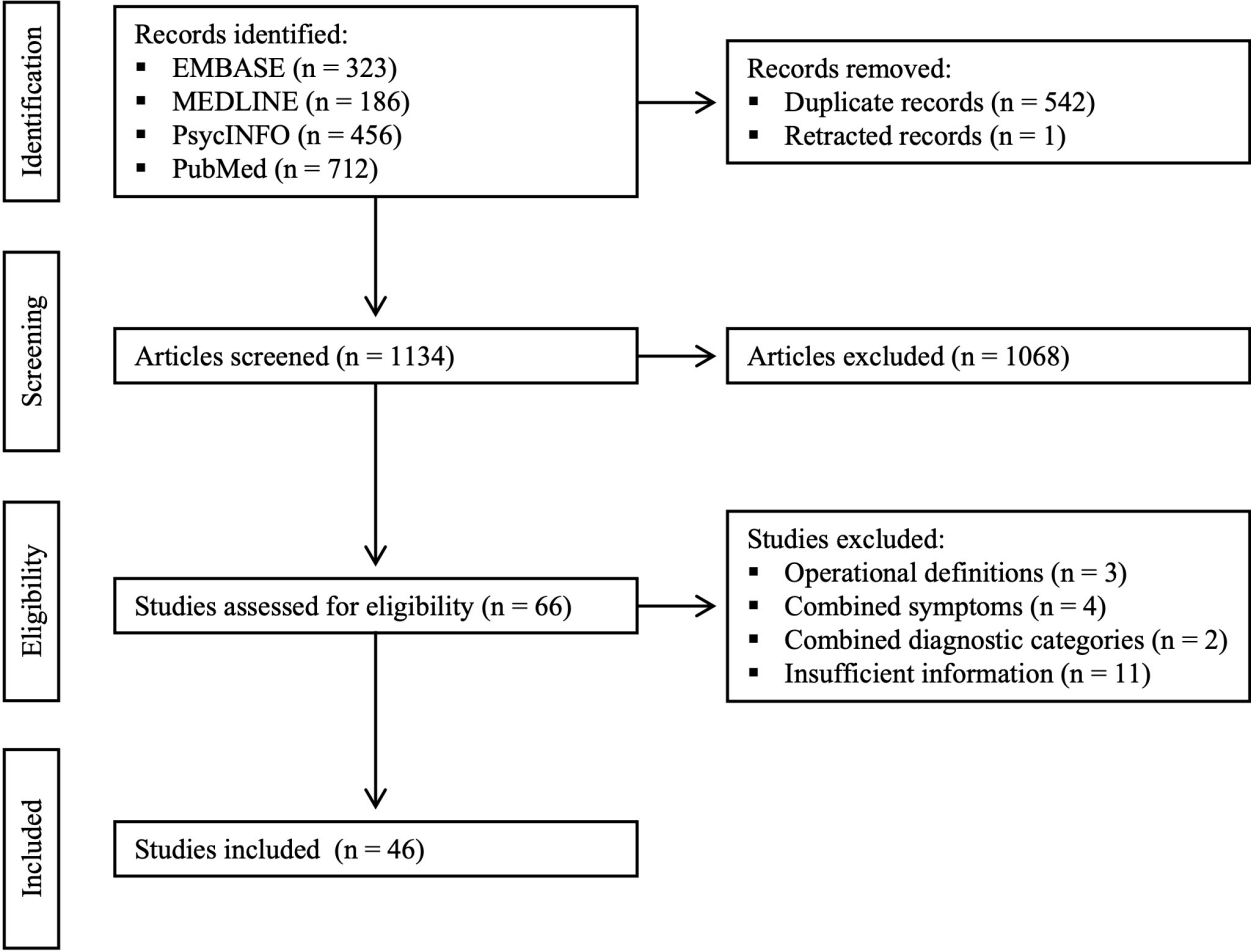
PRISMA flow diagram.

Database searching identified 1,677 records. After removing 542 duplicate records and one retracted record, 1,134 articles were screened by title and abstract. Then, after removing 1,068 irrelevant articles, 66 were assessed for eligibility by full text. Finally, 46 studies met the inclusion criteria, while 20 studies were excluded due to operational definitions (32–34), combined symptoms (35–38), combined diagnostic categories (11, 39), or insufficient information (40–50) (see Figure 1 for the PRISMA flow diagram).

### 3.2 Study characteristics

This systematic review and meta-analysis comprise data collected from 13 countries (Australia, Canada, China, Czech Republic, France, Germany, Italy, Japan, Mexico, Russia, Switzerland, United Kingdom, and United States), utilizing four different study designs (cohort, cross-sectional, case–control, and case report). The studies included healthy individuals and epilepsy patients that participated in interventions/investigations (surveys, interviews, neuroimaging techniques, and performance-based neurocognitive assessments), providing phenomenological and neuroscientific outcomes (see Table 1 for details).

**Table 1:** Study characteristics and quality assessment.

Study	Characteristics						Quality criteria					
	Country	Design	P	I	C	O	1	2	3	4	5	%
Adachi et al. (51)	United Kingdom	cohort	TLE w/iDV; *n* = 14; sex: 7 m/7 f; age: mean = 27, sd = 10 y	EEG, MRI, ^18^FDG PET	n/a	iDV – hemispheric laterality of seizure focus, CGM	1	1	1	1	1	100
			TLE w/o iDV; *n* = 17; sex: 7 m/10 f; age: mean = 30, sd = 10 y									
Adachi et al. (52)	Japan	cross-sectional	n/a	IDEA – part A (self-report inventory)	H; *n* = 386; sex: 188 m/198 f; age: mean = 38, sd = 12, range = 18–69 y	niDV – prevalence, sex, age, derealization, depersonalization	1	1	1	?	1	80
Adachi et al. (53)	Japan	cross-sectional	n/a	IDEA – part A (self-report inventory), DES (self-report questionnaire)	H; *n* = 227; sex: 99 m/128 f; age: mean = 40, sd = 14, range = 16–69 y	niDV – prevalence, sex, age, derealization, depersonalization, dissociation	1	1	1	?	1	80
Adachi et al. (18)	Japan	case–control	E; *n* = 312; sex: 164 m/148 f; age: mean = 35, sd = 11, range = 17–66 y	IDEA – part A and B (self-report inventory)	H; see Adachi et al. (52, 53)	i/iiDV – prevalence, sex, age, duration, affect, derealization, depersonalization	1	1	1	?	1	80
Bartolomei et al. (54)	France	cohort	E; *n* = 24; sex: n/a; age: n/a	electrical cortical stimulation, SEEG, seizure semiology	n/a	induced iDV – brain structures	1	1	1	?	1	80
Bartolomei et al. (55)	France	cohort	E; *n* = 7; sex: 4 m/3 f; age: mean = 33, sd = 9, range = 23–46 y	electrical cortical stimulation, SEEG, seizure semiology	n/a	induced iDV – brain structures	1	1	1	1	1	100
Brázdil et al. (56)	Czech Republic	cohort	n/a	SBM	H w/niDV; *n* = 87; sex: 45 m/42 f; age: mean = 25, sd = 4 y	niDV – GMV	1	1	1	1	1	100
					H w/o niDV; *n* = 26; sex: 13 m/13 f; age: mean = 26, sd = 7 y							
Carlson et al. (57)	United States	cohort	E; *n* = 538; sex: 260 f/278 m; age: n/a	seizure semiology (semi-structured interview)	n/a	iDV – prevalence, sex	1	1	1	0	1	80
Chen et al. (58)	United States	cohort	E; *n* = 9,221; sex: 3419 m/5802 f; age: median = 53, range = 35–67 y	ambulatory video EEG, seizure semiology (anamnesis)	n/a	iDV – incidence-epileptiform activity	1	1	1	1	1	100
Chong & Dugan (59)	United States	cohort	E; *n* = 512; sex: 249 m/263 f; age: mean = 23, sd = 16, range = 1–82 y	DI (semi-structured interview)	n/a	iDV – prevalence, fear	1	1	1	0	1	80
Cole & Zangwill (60)	United Kingdom	cohort	TLE w/iDV; *n* = 13; sex: n/a; age: n/a	n/a	n/a	iDV – hemispheric laterality of seizure focus	1	1	?	1	1	80
Crompton et al. (61)	Australia	cohort	FMTLE; *n* = 51; sex: 17 m/34 f; age: n/a	seizure semiology	n/a	iDV – prevalence	1	1	1	1	1	100
Curot et al. (62)	France	case report	TLE w/amnesia w/i/iiDV; *n* = 1; sex: f; age: 38 y	WMS-III, seizure semiology (anamnesis)	n/a	i/iiDV – recognition memory	1	?	1	1	?	60
Fortier & Moulin (63)	France	cross-sectional	n/a	phenomenology (self-report questionnaire)	H (English); *n* = 137; sex: 66 m/71 f; age: mean = 28, sd = 10 y	niDV – prevalence	1	1	1	?	1	80
					H (French); *n* = 456; sex: 87 m/369 f; age: 26, sd = 10 y							
Gelisse et al. (64)	France	case report	RE (musicogenic) w/iDV; *n* = 1; sex: f; age: 39 y	epileptogenic music, EEG, SPECT, seizure semiology	n/a	induced iDV – CBF	1	?	1	?	1	60
Guedj et al. (65)	France	case–control	TLE w/iDV; *n* = 8; sex: 2 m/6 f; age: mean = 37, sd = 14 y	^18^FDG PET	H; *n* = 20; sex: n/a; age: mean = 36, sd = 10 y	iDV – CGM	1	1	1	1	1	100
			TLE w/o iDV; *n* = 8; sex: 4 m/4 f; age: mean = 36, sd = 12 y									
Guzmán-Jiménez et al. (66)	Mexico	cohort	FMTLE; *n* = 61; sex: 30 m/31 f; age: mean = 26, sd = 16, range = 7–71 y	seizure semiology	n/a	iDV – prevalence	1	1	1	1	1	100
Halgren et al. (67)	United States	cohort	E; *n* = 36; sex: 23 m/13 f; age: mean = 26, sd = 8, range = 10–44 y	electrical cortical stimulation, seizure semiology	n/a	induced iDV – brain structures	1	1	1	1	1	100
Heydrich et al. (68)	Switzerland	cohort	TLE w/iDV; *n* = 16; sex: 7 m/9 f; age: mean = 32, sd = 12 y	multimodality imaging	n/a	iDV - hemispheric laterality of seizure focus	1	1	1	1	1	100
Kaaden et al. (69)	Germany	cohort	GAD+ AE; *n* = 20; sex: n/a; age: n/a	seizure semiology	n/a	iDV – prevalence	1	1	1	0	1	80
			LGI1+ AE; *n* = 59; sex: n/a; age: n/a									
			NMDAR+ AE; *n* = 51; sex: n/a; age: n/a									
Kerr et al. (70)	United States	cohort	E; *n* = 241; sex: n/a; age: n/a	seizure semiology (structured interview)	n/a	iDV – prevalence	1	1	1	0	1	80
Labate et al. (71)	Italy	case–control	MTLE w/iDV; *n* = 32; sex: 11 m/21 f; age: mean = 37, sd = 11, range = 20–57 y	VBM	H w/niDV; *n* = 22; sex: 8 m/14 f; age: mean = 34, sd = 8, range = 20–49 y	iDV/niDV – GMV	1	1	1	1	1	100
			MTLE w/o iDV; *n* = 31; sex: 11 m/20 f; age: mean = 39, sd = 10, range = 23–60 y		H w/o niDV; *n* = 17; sex: 9 m/8 f; age: mean = 35, sd = 8, range = 27–49 y							
Labate et al. (14)	Italy	case–control	E; *n* = 457; sex: 197 m/260 f; age: mean = 39, sd = 14 y	IDEA – part A and B (self-report inventory)	H; see Mumoli et al. (8)	iDV/iiDV – prevalence, sex, age, duration, affect, derealization, depersonalization	1	1	1	?	1	80
Lacinová et al. (72)	Czech Republic	cross-sectional	n/a	IDEA – part A (self-report inventory)	H; *n* = 365; sex: 147 m/218 f; age: mean = 29, sd = 11, range = 18–70 y	niDV – prevalence, sex, age	1	1	1	0	1	80
Levin (73)	United States	cross-sectional	n/a	GSS (self-report survey)	H; *n* = 1,456; sex: 628 m/828 f; age: mean = 46 y	niDV – prevalence, age	1	1	1	0	1	80
Martin et al. (74)	Canada	case–control	uTLE w/iDV; *n* = 7; sex: 2 m/5 f; age: mean = 28, sd = 9, range = 21–44 y	Remember-Know task, Exclusion task	H; *n* = 26; sex: 11 m/15 f; age: mean = 34, sd = 12 y	iDV – recognition memory	1	1	1	1	1	100
			uTLE w/o iDV; *n* = 6; sex: 2 m/4 f; age: mean = 36, sd = 9, range = 21–46 y									
Martin et al. (75)	Canada	case–control	uTLE w/iDV; see Martin et al. (74)	Remember-Know task, Exclusion task	H; see Martin et al. (74)	iDV – recognition memory	1	1	1	1	1	100
			bTLE w/iDV; *n* = 4; sex: 2 m/2 f; age: mean = 27, sd = 14, range = 19–48 y									
McClenon (7)	China	cross-sectional	n/a	UEQ (self-report questionnaire)	H; *n* = 314; sex: n/a; age: n/a (students)	niDV – prevalence	1	1	1	0	1	80
McClenon (76)	United States	cross-sectional	n/a	UEQ (self-report questionnaire)	H; *n* = 1,608; sex: n/a; age: n/a (students/scientists)	niDV – prevalence	1	1	1	0	1	80
Mumoli et al. (8)	Italy	cross-sectional	n/a	IDEA – part A and B (self-report inventory)	H; *n* = 542; sex: 232 m/310 f; age: mean = 40, sd = 20 y	niDV – prevalence, age, frequency, duration, affect, derealization, depersonalization	1	1	1	?	1	80
Nigro et al. (77)	Italy	cohort	n/a	fMRI tasks	H w/niDV; *n* = 18; sex: 10 m/8 f; age: mean = 28, sd = 5 y	niDV – recognition memory	1	1	1	1	1	100
					H w/o niDV; *n* = 15; sex: 5 m/10 f; age: mean = 31, sd = 10 y							
O’Connor and Moulin (78)	United Kingdom	cohort	n/a	Remember-Know tasks, phenomenology	H; *n* = 206; sex: 33 m/173 f; age: n/a (students)	niDV – incidence-recognition memory	1	1	1	1	1	100
Perucca et al. (79)	Australia	cohort	n/a	phenomenology (semi-structured interview)	H; *n* = 212; sex: n/a; age: n/a	niDV – prevalence	1	1	1	?	1	80
Pešlová et al. (80)	Czech Republic	case–control	L-MTLE; *n* = 27; sex: 8 m/19 f; age: median = 40, range = 18–55 y	MRI	H w/niDV; *n* = 87; sex: 45 m/42 f; age: median = 24, range = 19–46 y	niDV – GMV	1	1	1	1	1	100
			R-MTLE; *n* = 20; sex: 6 m/14 f; age: median = 38, range = 25–61 y		H w/o niDV; *n* = 26; sex: 14 m/12 f; age: median = 24, range = 20–50 y							
Qiu et al. (81)	China	cohort	n/a	VBM, rsFC	H w/niDV; *n* = 98; sex: 20 m/78 f; age: mean = 20, sd = 2 y	niDV – GMV, brain connectivity	1	1	1	1	1	100
Ross and Joshi (82)	United States	cross-sectional	n/a	DDIS (structured interview)	H; *n* = 502; sex: 184 m/318 f; age: mean = 45, sd = 17 y	niDV – prevalence	1	1	1	1	1	100
Shaw et al. (83)	Czech Republic	cohort	n/a	MRI	H; see Brázdil et al. (56)	niDV – brain structural covariance	1	1	1	1	1	100
Striano et al. (84)	Italy	cohort	FMTLE; *n* = 48; sex: 18 m/30 f; age: mean = 44, sd = 17, range = 10–85 y	seizure semiology	n/a	iDV – prevalence	1	1	1	1	1	100
Takeda et al. (85)	Japan	case report	TLE w/iDV; *n* = 1; sex; m; age: 51 y	SPECT/MRI	n/a	iDV – CBF	1	?	1	1	?	60
Toffa et al. (86)	Canada	case report	TLE w/iDV; *n* = 1; sex: m; age: 24 y	electrical cortical stimulation, video EEG, DTF, seizure semiology	n/a	induced iDV – brain structures	1	?	1	1	?	60
			TLE w/iDV; *n* = 1; sex: f; age: 32 y									
Van Paesschen et al. (87)	United Kingdom	cohort	TLE; *n* = 50; sex: 16 m/34 f; age: median = 31, range = 16–49 y	MRI (AT_2_), seizure semiology (standardized interview)	n/a	iDV – prevalence, brain structures	1	1	1	1	1	100
Vederman et al. (88)	United States	cohort	E w/mnemestic auras (88% w/iDV); *n* = 42; sex: 24 m/18 f; age: mean = 40, sd = 11, range = 18–60 y	CVLT	n/a	iDV – recognition memory	1	1	1	1	1	100
			E w/o mnemestic auras; *n* = 42; sex: 21 m/21 f; age: mean = 40, sd = 12, range = 19–61 y									
Vlasov et al. (89)	Russia	case report	E w/iDV; *n* = 1; sex: m; age: 29 y	ambulatory EEG, seizure semiology/ phenomenology	H w/niDV; *n* = 1; sex: f; age: 20 y	iDV/niDV – electrical brain activity	1	?	1	0	?	40
Weinand et al. (90)	United States	cohort	E w/iDV; *n* = 8; sex: 4 m/4 f; age: mean = 35, sd = 12, range = 20–54 y	ECoG	n/a	iDV – hemispheric laterality of seizure focus	1	1	1	1	1	100
Yang et al. (91)	China	cohort	OLE; *n* = 35; sex: 18 m/17 f; age: mean = 20, sd = 9, range = 4–37 y	seizure semiology	n/a	iDV – prevalence	1	1	1	1	1	100
Zatloukalova et al. (92)	Czech Republic	cohort	n/a	rs-fMRI (ALFF, fALFF)	H; *n* = 68 (65% w/niDV); sex: 38 m/30 f; age: mean = 26, sd = 4 y	niDV – brain activity	1	1	1	1	1	100

This table comprise study characteristics and quality assessment. The study characteristics include country, study design, population (P), intervention/investigation (I), control (C), and outcome (O). The quality assessment includes the following five quality criteria for quantitative descriptive studies: 1 = Is the sampling strategy relevant to address the research question? 2 = Is the sample representative of the target population? 3 = Are the measurements appropriate? 4 = Is the risk of nonresponse bias low? 5 = Is the statistical analysis appropriate to answer the research question? Quality criteria are scored with: 1 (“yes”), 0 (“no”), or ? (“cannot tell”). Quality criteria met (%): 1 = 20%, 2 = 40%, 3 = 60%, 4 = 80%, or 5 = 100%.AE, autoimmune encephalitis; ALFF, amplitude of low-frequency fluctuations; AT_2_, amygdala T_2_-weighted image; bTLE, bilateral temporal lobe epilepsy; CBF, cerebral blood flow; CGM, cerebral glucose metabolism; CVLT, California Verbal Learning Test; DDIS, Dissociative Disorders Interview Schedule; DES, Dissociative Experiences Scale; DI, Diagnostic Interview; DTF, directed transfer function; E, epilepsy; ECoG, electrocorticography; EEG, electroencephalography; f, female(s); fALFF, functional amplitude of low-frequency fluctuations; ^18^FDG PET, fluorine-18 fluorodeoxyglucose positron emission computed tomography; fMRI, functional magnetic resonance imaging; FMTLE, familial medial temporal lobe epilepsy; GAD+, glutamic acid decarboxylase antibody; GMV, gray matter volume; GSS, General Social Survey; H, healthy; IDEA, Inventory for Déjà vu Experiences Assessment; iDV, ictal déjà vu; iiDV, interictal déjà vu; LGI1+, leucine-rich glioma-inactivated protein 1 antibody; L-MTLE, left sided medial temporal lobe epilepsy; m, male(s); MRI, magnetic resonance imaging; MTLE, medial temporal lobe epilepsy; *n*, sample size; n/a, not available/applicable; niDV, non-ictal déjà vu; NMDAR+, N-methyl-D-aspartate receptor antibody; OLE, occipital lobe epilepsy; RE, reflex epilepsy; R-MTLE, right sided medial temporal lobe epilepsy; rsFC, resting-state functional connectivity; rs-fMRI, resting-state functional magnetic resonance imaging; sd, standard deviation; SEEG, stereoelectroencephalography; SBM, source-based morphometry; SPECT, single-photon emission computed tomography; TLE, temporal lobe epilepsy; UEQ, Unusual Experiences Questionnaire; uTLE, unilateral temporal lobe epilepsy; VBM, voxel-based morphometry; w/, with; WMS-III, Wechsler Memory Scale – Third edition; w/o, without; y, years.

### 3.3 Study outcomes

#### 3.3.1 Phenomenological aspects

##### 3.3.1.1 Non-ictal déjà vu

**Figure 2:**
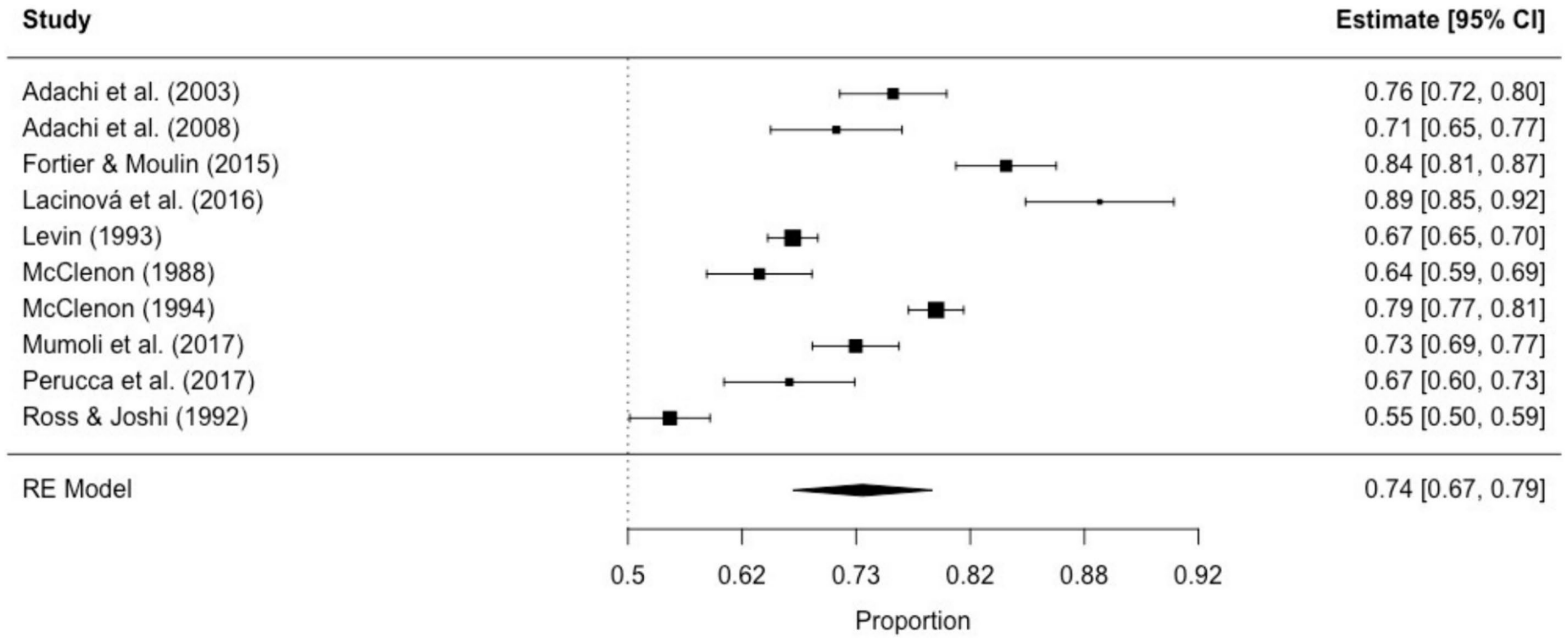
Forest plot of non-ictal déjà vu prevalence in healthy individuals. This plot illustrates study-specific proportions (squares) with corresponding weight (size of squares) and precision (error bars) alongside the pooled estimate from the random-effects (RE) model (diamond) with corresponding weight (diamond size) and precision (diamond width). Proportions have been back-transformed from logit.

**Figure 3:**
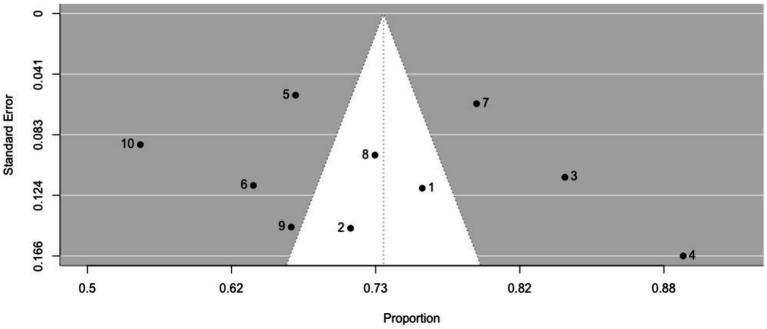
Funnel plot of non-ictal déjà vu prevalence in healthy individuals. This plot illustrates study-specific data points (numbered squares; 1 (52); 2 (53); 3 (63); 4 (72); 5 (73); 6 (7); 7 (76); 8 (8); 9 (79); 10 (82)) alongside the pooled estimate from the random-effects (RE) model (dotted line) plotted against proportions on the x-axis and standard error on the y-axis. Proportions have been back-transformed from logit.

The prevalence of non-ictal déjà vu in healthy individuals, estimated by a random-effects model combining 10 studies (7, 8, 52, 53, 63, 72, 73, 76, 79, 82), revealed a pooled proportion of 0.74 (95% CI [0.67, 0.79], *p* < 0.001; see Figure 2 for the forest plot). Considerable heterogeneity was observed among the study proportions (*I^2^* = 96.14%, *Q* (df = 9) = 233.23, *p* < 0.001), suggesting inconsistency across the studies. However, Egger’s regression test did not show asymmetry in the meta-analysis (*t*(df = 8) = 0.49, *p* = 0.637; see Figure 3 for the funnel plot), indicating no evidence of publication bias.

Non-ictal déjà vu correlated inversely with age (8, 52, 53, 72, 73), and was not associated with sex (52, 53, 72). The frequency of non-ictal déjà vu was reported weekly by 0.9%, a few times a month by 6.8%, a few times per year by 42.8%, or less than once per year by 22.5% (8). Each non-ictal déjà vu episode lasted 1 sec or less (8.9%), a few seconds (66.1%), 1 minute or a couple of minutes (19.5%), half an hour to 1 h (0.8%), a few hours (0.3%), or more than a few hours (0.3%) (8). Most participants experienced the non-ictal déjà vu episodes as emotionally indifferent or positive (e.g., reassuring, pleasant, and surprising), while some experienced them as emotionally negative (e.g., alarming, oppressing, and disturbing) (8). Co-occurring derealization correlated with non-ictal déjà vu, while data on depersonalization showed no consensus (8, 52, 53). Nevertheless, dissociation was not associated with non-ictal déjà vu (53).

##### 3.3.1.2 Interictal déjà vu

**Figure 4:**
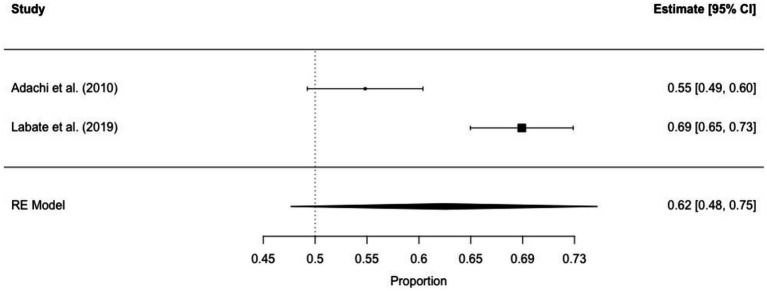
Forest plot of interictal déjà vu prevalence in epilepsy patients. This plot illustrates study-specific proportions (squares) with corresponding weight (size of squares) and precision (error bars) alongside the pooled estimate from the random-effects (RE) model (diamond) with corresponding weight (diamond size) and precision (diamond width). Proportions have been back-transformed from logit.

The prevalence of interictal déjà vu in epilepsy patients, estimated by a random-effects meta-analysis combining two studies (14, 18), revealed a pooled proportion of 0.62 (95% CI [0.48, 0.75], *p* = 0.099; see Figure 4 for the forest plot). Considerable heterogeneity was observed among the study proportions (*I^2^* = 93.65%, *Q* (df = 1) = 15.75, *p* < 0.001), suggesting inconsistency across the studies. Due to the limited number of studies included, Egger’s regression test for assessing publication bias in the meta-analysis was not performed.

Interictal déjà vu correlated inversely with age (14, 18), and was not associated with sex (14, 18). Compared to non-ictal déjà vu in healthy controls, interictal déjà vu was reported lasting equally long, more emotionally negative, and with higher co-occurrence of derealization and depersonalization (14).

##### 3.3.1.3 Ictal déjà vu

**Figure 5:**
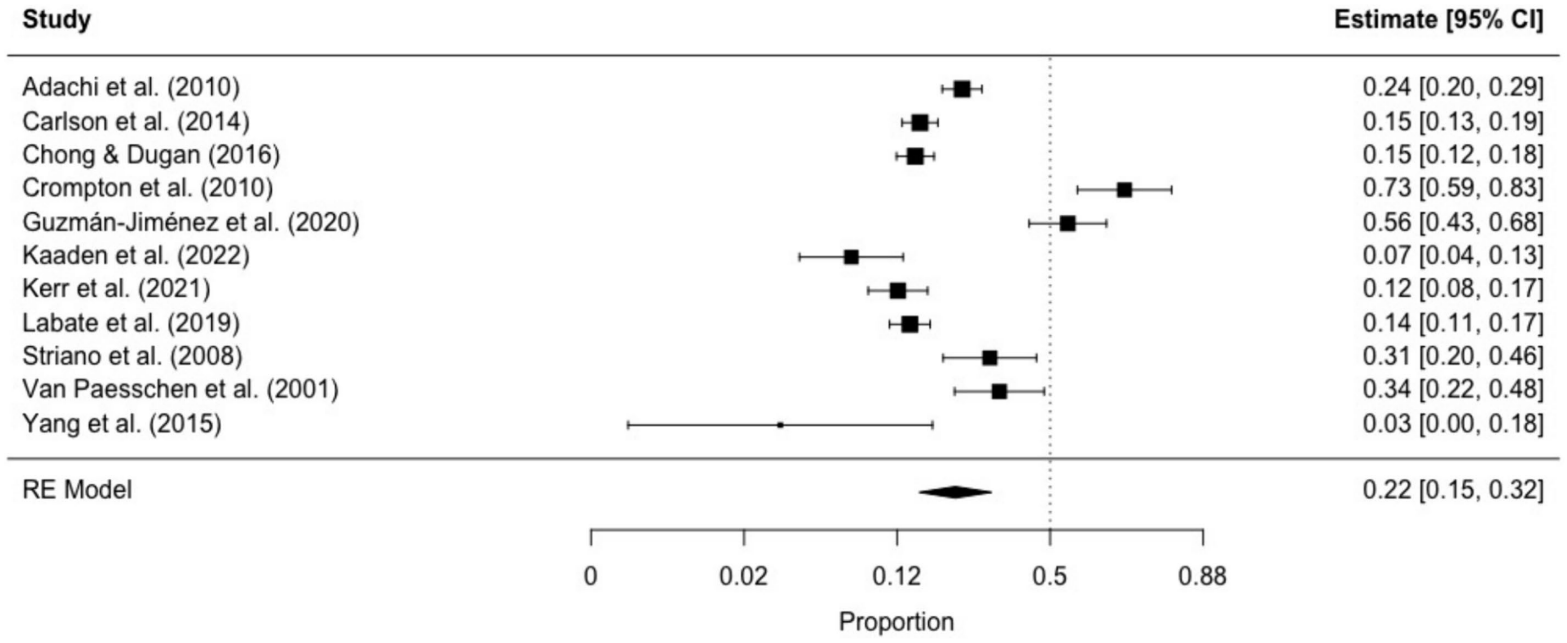
Forest plot of ictal déjà vu prevalence in epilepsy patients. This plot illustrates study-specific proportions (squares) with corresponding weight (size of squares) and precision (error bars) alongside the pooled estimate from the random-effects (RE) model (diamond) with corresponding weight (diamond size) and precision (diamond width). Proportions have been back-transformed from logit.

**Figure 6:**
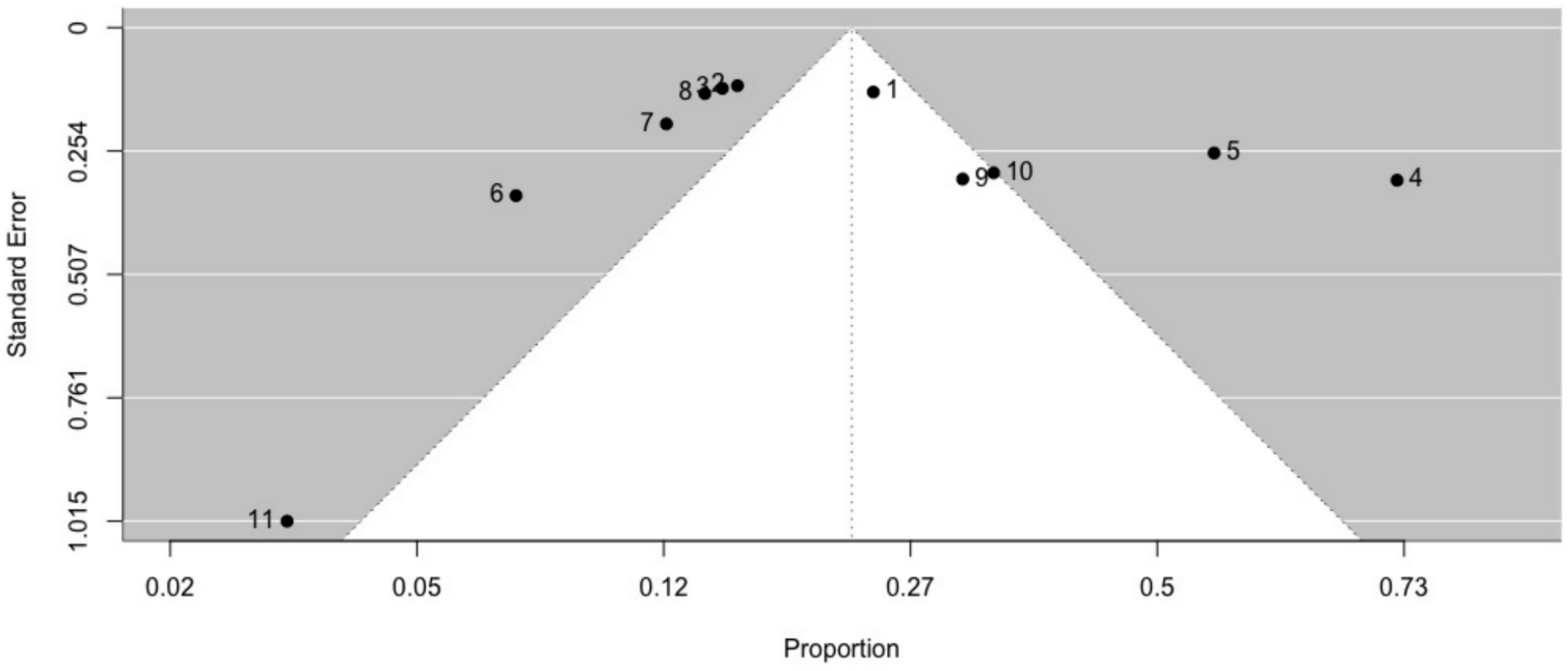
Funnel plot of ictal déjà vu prevalence in epilepsy patients. This plot illustrates study-specific data points (numbered squares; 1 (18); 2 (57); 3 (59); 4 (61); 5 (66); 6 (69); 7 (70); 8 (14); 9 (84); 10 (87); 11 (91)) alongside the pooled estimate from the random-effects (RE) model (dotted line) plotted against proportions on the x-axis and standard error on the y-axis. Proportions have been back-transformed from logit.

The prevalence of ictal déjà vu in epilepsy patients, estimated by a random-effects meta-analysis combining 11 studies (14, 18, 57, 59, 61, 66, 69, 70, 84, 87, 91), revealed a pooled proportion of 0.22 (95% CI [0.15, 0.32], *p* < 0.001; see Figure 5 for the forest plot). Considerable heterogeneity was observed among the study proportions (*I^2^* = 93.75%, *Q* (df = 10) = 159.96, *p* < 0.001), suggesting inconsistency across the studies. However, Egger’s regression test did not show asymmetry in the meta-analysis (*t* (df = 9) = 1.01, *p* = 0.335; see Figure 6 for the funnel plot), indicating no evidence of publication bias. The prevalence of ictal déjà vu was highest in familial medial temporal lobe epilepsy (FMTLE) (61) and lowest in occipital lobe epilepsy (OLE) (91).

Ictal déjà vu did not correlate with age (18), whereas data on sex showed no consensus (18, 57). Compared to interictal déjà vu in epilepsy patients, ictal déjà vu was reported lasting equally long, more emotionally negative, and with higher co-occurrence of derealization and depersonalization (18). Notably, ictal déjà vu correlated with fear (59).

#### 3.3.2 Brain structures and functions

##### 3.3.2.1 Non-ictal déjà vu

Electrical brain activity, monitored in a healthy individual during non-ictal déjà vu episodes through ambulatory electroencephalography (EEG), revealed no epileptiform activity, but noted rhythm desynchronization (89). Further investigations, using resting-state functional magnetic resonance imaging (rs-fMRI), identified significant reductions in amplitude of low-frequency fluctuations (ALFF) in the superior frontal cortex and parahippocampal gyrus, as well as in fractional ALFF (fALFF) in the caudate, putamen, amygdala, and thalamus, among participants experiencing non-ictal déjà vu compared to those without such experiences (92). Moreover, the frequency of non-ictal déjà vu correlated negatively with the resting-state functional connectivity (rsFC) strenght between the anterior parts of the dorsolateral prefrontal cortex and parahippocampal gyrus, yet positively between the posterior parts of the dorsolateral prefrontal cortex and parahippocampal gyrus (81).

Source-based morphometry (SBM) in healthy individuals experiencing non-ictal déjà vu revealed a reduction in gray matter volume (GMV) within several brain regions, including the insula, parahippocampal gyrus, superior temporal sulcus, thalamus, basal ganglia, and hippocampus, compared to participants without such experiences. This reduction correlated inversely with non-ictal déjà vu frequency (56). Additionally, brain regions with higher non-ictal déjà vu frequency demonstrated specific patterns of GMV covariance (83). Voxel-based morphometry (VBM) revealed that participants experiencing non-ictal déjà vu weekly or monthly had lower GMV in the anterior parahippocampal gyrus than those experiencing it yearly. Conversely, those with a higher frequency of non-ictal déjà vu exhibited more significant GMV in the superior frontal and temporal gyrus than those with a lower frequency (81). No differences were found in hippocampal GMV among individuals with non-ictal déjà vu, those without, and MTLE patients (80).

Assessment of recognition memory in healthy individuals showed that non-ictal déjà vu did not predict familiarity or recollection memory performance on fMRI tasks (77), nor did the frequency of non-ictal déjà vu correlate with familiarity or recollection parameters on Remember-Know tasks (78). However, independent of the fMRI task performance, distinct brain region activities varied between the two groups. Specifically, enhanced activation in the insula and decreased activation in the superior frontal, medial temporal, and parahippocampal gyrus were observed in individuals with non-ictal déjà vu relative to those without (77).

##### 3.3.2.2 Ictal déjà vu

Electrical brain activity, monitored in epilepsy patients during ictal déjà vu episodes through ambulatory video EEG, revealed epileptiform activity (58, 89). Further examinations showed that, the hemispheric laterality of the seizure focus did not significantly influence the occurrence of ictal déjà vu (51, 60, 68, 90). Electrical stimulation through implanted cortical electrodes in drug-resistant epilepsy patients highlighted the involvement of specific brain regions during stimuli-induced ictal déjà vu episodes, including the insula (86), rhinal cortex (54, 55), parahippocampal gyrus (67), hippocampus (54, 67), and amygdala (54, 67). Observations showed that the frequency of stimuli-induced ictal déjà vu episodes was significantly higher in the entorhinal cortex compared to the perirhinal cortex, and in the rhinal cortex relative to the hippocampus and amygdala (54). However, as these observations were made as part of a pre-surgical procedure and none of these studies reported post-surgical data, it is unknown how seizure outcomes (e.g., defined by the Engel Epilepsy Surgery Outcome Scale) affected the frequency of ictal déjà vu episodes. Additionally, functional connectivity, examined through stereoelectroencephalography (SEEG) broadband signals in drug-resistant epilepsy patients experiencing stimuli-induced ictal déjà vu episodes, indicated increased activity. This was especially evident in the enhanced interaction between the rhinal cortex and hippocampus, and between the rhinal cortex and amygdala, distinguishing stimuli-induced ictal déjà vu episodes from non-stimuli-induced states (55).

VBM, assessed in MTLE patients with and without ictal déjà vu, as well as in healthy controls with and without non-ictal déjà vu, revealed no significant differences in GMV across the groups (71). However, MRI, assessed in temporal lobe epilepsy (TLE) patients, indicated that the occurrence of ictal déjà vu serves as a reliable predictor of amygdala abnormalities (87).

Cerebral blood flow (CBF), measured in epilepsy patients with ictal déjà vu using single-photon emission computed tomography (SPECT), showed hyperperfusion in the temporal lobe (64) and entorhinal cortex (85) during ictal déjà vu episodes. Cerebral glucose metabolism (CGM), measured in TLE patients with and without ictal déjà vu using fluorine-18 fluorodeoxyglucose positron emission computed tomography (^18^FDG-PET), demonstrated hypometabolism in the parietal cortex and medial temporal lobe in those with ictal déjà vu compared to those without (51). Further analyses indicated hypometabolism in the superior temporal gyrus and parahippocampal region in TLE patients with ictal déjà vu relative to those without and healthy controls (65).

Visual memory, assessed in TLE patients, with and without ictal déjà vu, and healthy controls using Remember-Know and Exclusion tasks, showed that ictal déjà vu does not impact recognition abilities (74, 75). Additionally, a case report of a bilateral TLE patient with hippocampal lesions and severe episodic amnesia was noted to encounter both ictal and interictal déjà vu (62). Verbal memory, evaluated in epilepsy patients using the California Verbal Learning Test (CVLT), indicated that ictal déjà vu does not predict CVLT performance outcomes (88).

### 3.4 Study risk of bias assessment

The study risk of bias assessment, using the MMAT version 18 (23, 24), showed that out of the 46 studies evaluated, 46 studies (100%) satisfied the relevance of the sampling strategy criterion, 41 studies (89.1%) met the criterion for sample representativeness, 45 (97.8%) complied with the appropriateness of measurements criterion, 28 studies (60.9%) were considered to have a low risk of nonresponse bias, and 42 studies (91.3%) fulfilled the criterion for appropriateness of statistical analysis. In terms of overall methodological quality, 24 studies (52.2%) met all the criteria (100%), 17 studies (37.0%) satisfied 80% of the criteria, four studies (8.7%) met 60% of the criteria, and one study (2.2%) satisfied 40% of the criteria (see Table 1 for details).

## 4 Discussion

This systematic review and meta-analysis include 46 empirical studies examining phenomenological and neuroscientific aspects of non-ictal, interictal and ictal déjà vu, insights that may have clinical implications.

### 4.1 Comparison and interpretation of the main findings

Evidence suggests variations in the phenomenology of non-ictal, interictal, and ictal déjà vu experiences. Non-ictal déjà vu emerged as the most prevalent, followed by interictal déjà vu, and ictal déjà vu was the least prevalent. The duration of these episodes remained consistent across all phenomena. Both non-ictal and interictal déjà vu correlated inversely with age, a trend not observed in ictal déjà vu. Similarly, both non-ictal and interictal déjà vu were not associated with sex, whereas data on sex showed no consensus in ictal déjà vu. Emotionally, non-ictal déjà vu was associated with less negative affect, followed by interictal déjà vu, and ictal déjà vu was associated with the most negative affect, particularly manifesting as fear. Regarding dissociation, non-ictal déjà vu did not exhibit a correlation. However, the co-occurrence of derealization and depersonalization was more pronounced in interictal déjà vu compared to non-ictal déjà vu, and even more so in ictal déjà vu compared to interictal déjà vu.

Additionally, differences in brain structures and functions between ictal and non-ictal déjà vu experiences were unveiled. Epileptiform activity was recorded during ictal déjà vu episodes, while it was absent during non-ictal déjà vu episodes. Further examination revealed that the hemispheric laterality of the seizure focus did not affect the occurrence of ictal déjà vu. Moreover, electrical stimulation through implanted cortical electrodes highlighted the involvement of specific brain regions during stimuli-induced ictal déjà vu episodes, with the highest frequency reported in the rhinal cortex, followed by the amygdala and hippocampus. Alterations in brain connectivity were also observed, with increased connectivity between the rhinal cortex and hippocampus, as well as between the rhinal cortex and amygdala in ictal déjà vu. In contrast, no clear pattern emerged in non-ictal déjà vu. Furthermore, analyses of GMV produced inconsistent results for ictal and non-ictal déjà vu, while changes in CBF and CGM were distinctive features of ictal déjà vu. Neither ictal nor non-ictal déjà vu predicted performance on recognition memory tasks.

These findings do not support the notion that non-ictal, interictal and ictal déjà vu are homogenous experiences. Instead, they suggest a classification into non-pathological (non-ictal and interictal déjà vu) and pathological (ictal déjà vu) categories based on distinct characteristics. Non-ictal and interictal déjà vu are more prevalent, inversely correlated with age, and are associated with less negative affect and dissociative symptoms than their ictal counterpart. The impact of age on these phenomena initially suggested a potential explanation rooted in brain aging (93), which typically involves declining brain connectivity and cognitive function (94). However, this hypothesis does not fully account for the observed age gap, as non-ictal déjà vu does not show impairments in recognition memory. Unlike ictal déjà vu, non-ictal déjà vu is not associated with epileptiform discharges but instead shows rhythm desynchronization, highlighting its non-pathological characteristics.

Ictal déjà vu emerges as a distinct pathological symptom that occurs exclusively during epileptic seizures. Despite its specificity, it is not universally experienced by epilepsy patients; rather, it is primarily localized to the medial temporal lobe and is most frequently reported in cases of FMTLE. Although ictal déjà vu is associated with epileptiform activity, this association should not be considered the norm. Indeed, some seizures, particularly those involving “epileptic auras,” may exhibit a normal EEG pattern (95, 96). Additionally, persistent (ictal) déjà vu can precede an epilepsy diagnosis by months (97) or even years (98), potentially serving as a prodromal sign of the brain disease. Unlike non-ictal and interictal déjà vu, ictal déjà vu is associated with more negative affect, something that is likely tied to the amygdala’s role in fear processing (99). This is underscored by increased functional connectivity observed between the entorhinal cortex and amygdala, as well as between the entorhinal cortex and hippocampus during ictal déjà vu episodes, suggesting that ictal déjà vu results from the interplay within and between brain networks rather than being attributable to a single brain structure. Ictal déjà vu is further characterized by changes in CBF and CGM, both of which can serve as indicators of abnormal neuronal activity associated with epilepsy. Hyperperfusion during seizures is believed to reflect the increased energy demands of excessive or synchronous neuronal activity; conversely, hypometabolism between seizures is thought to represent the aftermath of these high energy demands (100). In terms of visual and verbal memory, ictal déjà vu does not seem to be associated with recognition abilities, supporting the idea that perceptual shifts of focus during ictal déjà vu episodes do not diminish the experience (101). Together, these insights into ictal déjà vu underscore its pathological significance.

### 4.2 Study limitations

This systematic review and meta-analysis is comprehensive yet acknowledges its inherent limitations. First, all studies relied on accurate self-reporting of non-ictal, interictal and ictal déjà vu, introducing evident limitations because these are subjective experiences, not objective signs. Second, not all studies satisfied the methodological quality criteria for quantitative descriptive research. Most studies achieved an overall quality score between 80 and 100%. Those falling short often failed to address the risk of nonresponse bias. Few studies achieved an overall quality score between 40 and 60%. Those falling short did so mainly due to their nature as case reports, which limits their ability to generalize outcomes and perform statistical analyses. Third, meta-analyses were conducted solely on prevalence data. While some studies discussed their outcomes qualitatively, they did not provide numerical data, limiting the scope of our analysis. Incorporating partial data from each phenomenological parameter would have compromised the study’s overall quality. Across all meta-analyses, considerable heterogeneity was observed, likely attributed to the various study designs employed. However, no evidence of publication bias was detected.

### 4.3 Clinical implications and future directions

The inclusion of ictal déjà vu in diagnostic manuals has been previously discussed (102–105). However, up to this point, there has been a lack of evidence-based research to substantiate this proposal. This systematic review and meta-analysis provide support for considering ictal déjà vu as a symptom of epilepsy. Its inclusion in future revisions of the ICD-11 could potentially facilitate earlier interventions and improve outcomes for those predisposed to or diagnosed with epilepsy.

Further exploration is warranted regarding the predictive value of persistent (ictal) déjà vu experiences for epilepsy diagnosis. Prospective cohort studies should delve into the potential of (ictal) déjà vu, particularly when accompanied by negative affect, as a possible prodromal sign of the brain disease, as has been suggested previously (97). Such investigations would enrich our understanding of epilepsy onset and progression.

## Data availability statement

Publicly available datasets were analyzed in this study. The names of the repository/repositories and accession numbers can be found in the article.

## Author contributions

AH: Conceptualization, Data curation, Formal analysis, Investigation, Methodology, Validation, Visualization, Writing – original draft, Writing – review & editing. SA: Conceptualization, Data curation, Formal analysis, Investigation, Methodology, Supervision, Validation, Visualization, Writing – original draft, Writing – review & editing.
